# Epidemiology of Dry Eye and Its Determinants Among University Students

**DOI:** 10.18502/jovr.v17i3.11586

**Published:** 2022-08-15

**Authors:** Seyed Saber Sahih Alnasab, Amir Asharlous, Asgar Doostdar, Payam Nabovati, Abbasali Yekta, Mehdi Khabazkhoob

**Affiliations:** ^1^Noor Research Center for Ophthalmic Epidemiology, Noor Eye Hospital, Tehran, Iran; ^2^Rehabilitation Research Center, Department of Optometry, School of Rehabilitation Sciences, Iran University of Medical Sciences, Tehran, Iran; ^3^Department of Optometry, Mashhad University of Medical Sciences, Mashhad, Iran; ^4^Department of Basic Sciences, School of Nursing and Midwifery, Shahid Beheshti University of Medical Sciences, Tehran, Iran

##  Dear Editor,

Dry eye syndrome (DES) is defined as a multifactorial disease of the tears and ocular surface which is associated with symptoms of discomfort and visual disturbance, with potential damage to the ocular surface.^[1]^ Dry eye may cause several ocular and visual symptoms (including stinging or burning, excessive tearing, gritty sensation, episodes of blurred vision, and redness) negatively impacting the quality of life due to its extensive ocular consequences.^[1]^ About 60% of the patients complain about decreased quality of life in their daily and leisurely activities.^[2]^ University students comprise a young population that spends a significant amount of time on reading and working with the computer. The aim of this letter is to evaluate the prevalence of DES and its determinants in a population of Iranian university students. For this purpose, we carried out a cross-sectional study on 850 university students (407 male subjects) with a mean age of 22.06 ± 4 years. First, the subjects who met the inclusion criteria completed the Persian version of the standard Ocular Surface Disease Index (OSDI) questionnaire, and their sleep hours and duration of working with the computer as well as outdoor activities were recorded as monthly average. Next, the subjects underwent an ocular surface examination using a slit lamp to assess their ocular surface health. Then, the tear meniscus height (TMH) was measured and the Schirmer's test (without anesthesia), tear break-up time (TBUT), and fluorescein eye stain were done. A wash-out period was considered between tests. All subjects were examined in one room with similar conditions.

The overall prevalence of DES was 16.36%. Abnormal Schirmer's test ≤ 5, TBUT ≤ 10, and TMH ≤ 0.2 mm, OSDI ≥ 23, and fluorescein eye stain results were observed as 21.45%, 65.12%, 39.66%, 32.25%, and 35.44%, respectively. At least one symptom according to OSDI (sensitivity to light, gritty sensation, painful or sore eyes, blurred vision, poor vision) was always present in 15.25% of the cases. The prevalence of DES was 22.40% in female and 12.78% in male (odds ratio = 1.09, *P* = 0.032) students. There was no correlation between the prevalence of DES and age (*P* = 0.629). The prevalence of DES in different ethnic groups was not statistically significant (*P* = 0.094) and increased significantly with an increase in working with computers (*P* = 0.013). The prevalence of DES had no correlation with sleep hours (*P* = 0.155) and duration of outdoor activity (*P* = 0.593).

The prevalence of DES was higher in Iranian students compared to other similar studies, which could be due to geographical, lifestyle, climatic, and even ethnic differences.^[3]^ While some studies ${}^{\left[4,5\right]}$ reported a direct correlation between the prevalence of dry eye and age, this study found no significant relationship between DES prevalence and age, which could be due to the limited age range of the subjects. In this study, like most previous studies,^[4,5]^ the prevalence of DES was significantly higher in women than men, which is probably due to the hormonal changes, especially estrogen-related changes in women.^[6]^

This study revealed a significant correlation between hours of working on digital monitors per day and DES, which was consistent with previous studies.^[7]^The reason for this finding could be lower blinking during working with digital gadgets and the presence of the wide width of the palpebral fissure when working with computers.^[7]^

In summary, the results showed a higher prevalence of DES in young students as compared to the general population, which is in line with previous studies in literature. Female gender and increased computer working time are risk factors of DES in university students.

##  Ethics Approval

The Ethics Committee of Iran University of Medical Sciences approved the study protocol, which was conducted in accord with the tenets of the Helsinki Declaration. All participants signed a written informed consent (Iran University of Medical Sciences ethics approval: 965879).

##  Financial Support and Sponsorship

This project was supported by Iran University of Medical Sciences.

##  Conflicts of Interest

None.
